# Dextranomer/Hyaluronic Acid Calcification Masquerading as Distal Ureteral Calculi in a Patient Previously Treated for Vesicoureteral Reflux

**DOI:** 10.1089/cren.2017.0051

**Published:** 2018-04-01

**Authors:** Muammer Bozkurt, Samir Agalarov, Erkan Merder, Fatih Altunrende

**Affiliations:** Department of Urology, Okmeydani Training and Research Hospital, Istanbul, Turkey.

**Keywords:** vesicoureteral reflux, ureteral calculi, subureteral injection

## Abstract

Subureteral endoscopic injection is a safe and effective treatment for vesicoureteral reflux (VUR). Dextranomer/hyaluronic acid (Dx/HA) is the most commonly used bulking agent for the treatment of VUR. We report a confusing radiographic finding of calcified Dx/HA injection initially simulates distal ureteral stone in a female patient who has intermittent lumbar pain. Calcification of Dx/HA implants may mimic distal ureteral calculi; therefore, urologists should be aware of the potentially confusing radiographic images.

## Introduction

The prevalence of vesicoureteral reflux (VUR) in children has been estimated at 0.4%–1.8%. Subureteral endoscopic injection is a safe and effective treatment for VUR. Several bulking agents have been used for the past two decades. Currently, dextranomer/hyaluronic acid (Dx/HA) is the most popular bulking agent approved by the Food and Drug Administration in the treatment of VUR. Typical histologic changes, including calcification, are seen at the injection site after injection. These calcifications at the injection site can potentially lead to misdiagnosis.

We report an example of a confusing radiographic finding in a patient with a history of endoscopic treatment of VUR with Dx/HA 6 years ago.

## Case Summary

**FIG. 1.**
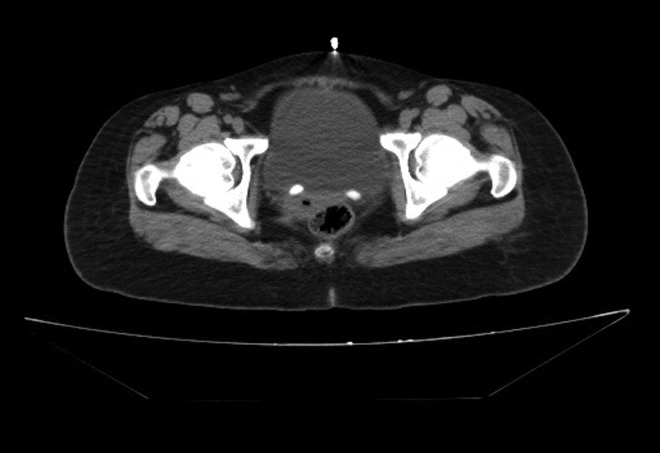
Computed tomography.

A 29-year-old female patient presented with complaint of intermittent right flank pain. She had mild hydronephrosis on ultrasonography. The patient was evaluated with CT. Abdominal CT image interpreted calcifications of a size of 12 mm in bilateral ureterovesical junction and mild right hydronephrosis and no left kidney (Fig. 1). The patient was referred to our hospital with prediagnosis of bilateral ureteral stones. But there was no stone appearance that conformed to the same localization in direct radiography. A review of the patient's medical history revealed that she had received bilateral endoscopic injections of Dx/HA for VUR (right: grade 2, left: grade 5) 6 years ago, followed by a left nephrectomy after a year.

**FIG. 2.**
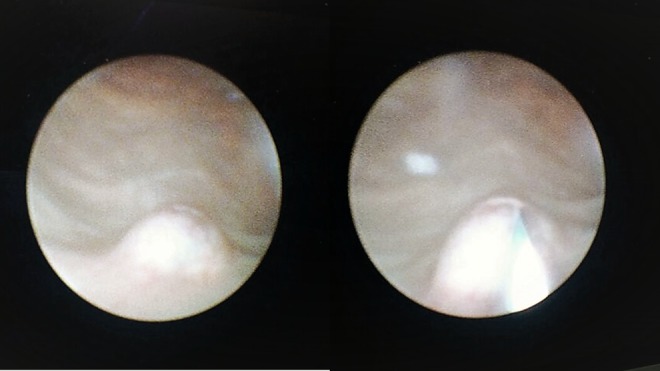
Cystoscopic image.

Urine analysis was normal and urine culture was sterile. After a thorough examination, ureteroscopy was planned, because the patient had a single kidney. We observed the volcanic cone-like area located at caudal of the bilateral orifices in the cystoscopy (Fig. 2). Ureteroscopy showed no stone in ureters. The patient was discharged. Follow-up after 3 months CT showed no change in calcification at the ureterovesical junction.

## Discussion

Many different molecules have been used in the historical process for injection of VUR. Dx/HA is the molecule that approaches the optimal injection material most probably because of the lack of migration and bulk reduction, its biocompatibility, and its high treatment success in studies. For these reasons, its present use is almost standardized.

Endoscopic injection of Dx/HA that is well standardized is relatively safe. This treatment has minimal complication rates. Histologic changes can develop after injection of Dx/HA, including calcification at the injection site after several years.

The reason for calcification of Dx/HA is unclear. Different theories have been proposed, including localized hypercalcemia, the precipitation of calcium salts from the Dx/HA formulation, microbial infection, or as a result of the inflammatory reaction.^1^

To assess the safety of Dx/HA, histologic studies of the distal ureter at the site of injection have been conducted that revealed the formation of a pseudocapsule with granulomatous inflammation and evidence of postinjection calcification after a mean period of 22 months (13–39 months).^1^

Yankovic et al.^2^ reported that the incidence of Dx/HA calcification is about 2% in patients followed up for a minimum of 4 years. This study was evaluated using ultrasound in the follow-up after endoscopic therapy. In these patients, hyperechogenic foci with acoustic shadowing, reported as distal ureteral calculi, were observed on ultrasonography.

Cerwinka and coworkers reported that Dx/HA for endoscopic VUR in children is observed on CT as high-density or low-density implants. According to this study, elapsed time between surgery and CT was associated with increased implant density.^3^

The misinterpretation of calcified Dx–HA as ureteral stones was first reported in 2008.^4^ Similar cases have been reported in this case until now.

A lack of hydronephrosis and flank pain may help to distinguish calcified implants from ureteral calculi in those with VUR treatment. However, this may be difficult in patients who present with subjective pain and mild hydronephrosis. We planned to perform ureterorenoscopy for this patient who was referred to us from the external center because the patient had intermittent pain and mild hydronephrosis. This patient also had solitary kidney.

## Conclusions

As has been demonstrated in our report, a calcified area corresponding to previous Dx/HA injection can easily be mistaken for a distal ureteral stone. Physicians should be sensitive of the risk of calcification; if ureteral calcifications are detected in patients with a history of Dx/HA injections, care should be taken to investigate carefully and avoid unnecessary interventions.

## Abbreviations Used

CT: computed tomography
Dx/HA: dextranomer/hyaluronic acid
VUR: vesicoureteral reflux
